# Supplementary material for: Personality Characteristics of Children and Adolescents with Anxiety Disorder from a Maternal Perspective: A Brief Report
Source: Behav Sci (Basel). 2023 May 12;13(5):404. doi: 10.3390/bs13050404 (PMC10215878; doi:10.3390/bs13050404)
Supplement: Supplementary file 1 [file behavsci-13-00404-s001.zip › behavsci-2312793-supplementary.pdf]

behavioral sciences

MDPI

Personality characteristics of children and adolescents with anxiety disorder from a maternal perspective: a brief report

Back Matter

Table S1- Sociodemographic and clinical measures. Clinical group and control group.

Sociodemographic and clinical data	Total (n=48)	Clinical group (n=24)	Control group (n=24)	P value
**Child's age (years)** [1] M (SD)	12.75 (2.88)	12.42 (2.86)	13.08 (2.92)	0.43
**Educational level** [1] M (SD)	7.31 (2.78)	7.25 (2.88)	7.38 (2.75)	0.88
**IQ - Total** [1] M (SD)	103.63 (15.33)	96.33 (13.13)	110.92 (14.02)	<0.001
**Gender** [2] N (%)				
(female/male)	58.3 / 41.7	54.2 / 45.8	62.5 / 37.5	0.56
**Socioeconomic level** [2] N (%)				
A	18.8	0	37.5	
B - C	81.2	100	62.5	<0.001
D - E	0	0	0	
**Mother's educational level** [2] (%)				
Elementary School	18.8	33.3	4.2	
High school	29.2	50	8.3	<0.001
Higher education	52	16.7	87.5	
**Mother's age** [1] **M (SD)**	43.63 (5.89)	43 (6.65)	44.25 (5.1)	0.47
**Mother's marital status** [2] N (%)				
Single	10.4	12.5	8.3	
Married	79.2	83.3	75	0.35
Divorced	10.4	4.2	16.7	
Widow	0	0	0	
**Mother's SRQ** [1] M (SD)	4.4 (3.89)	6.08 (4.3)	2.71 (2.53)	<0.001
**Clinical assessment** [1] M (SD)				
CBCL (total of competences)	37.65 (13.82)	32.08 (8)	43.21 (16.18)	<0.001
CBCL Int. problems T>60	59.6 (14.39)	68.04 (13.56)	51.17 (9.51)	<0.001
CBCL Ext. problems T>60	50.96 (9.99)	55.29 (9.51)	46.63 (8.62)	<0.001
CBCL Anxiety (60 - 70)	62.67 (10.96)	69.13 (11.09)	56.21 (5.93)	<0.001
CBCL Depression (60 - 70)	59.71 (11.79)	67.04 (12.02)	52.38 (5.26)	<0.001
MASC-2				
Child	58.17 (12.29)	61.63 (13.33)	54.71 (10.29)	0.08
Mother	59.5 (16.15)	68.38 (15.71)	50.63 (11.02)	<0.001
EPQ-J				
Psychoticism	31.83 (29.09)	35.75 (29.77)	27.92 (28.40)	0.27
Extroversion	39.38 (27.18)	31.25 (22.81)	47.50 (29.19)	0.05
Neuroticism	54.83 (33.83)	61.00 (34.99)	48.67 (32.17)	0.22
Sincerity	39.27 (24.95)	38.54 (25.30)	40.00 (25.11)	0.92

Note: [1] Student's t-test / Mann-Whitney; [2] Chi-Square Test

Table S2 - Personality Inventory for Children. Second Edition (PIC-2). Measures of the clinical group versus the control group. Covariate IQ effect.

PIC-2	Clinical group *M* (*SD*)	Control group *M* (*SD*)	*p* value	*p* value ANCOVA (IQ)
**Response Validity Scales**				
Inconsistency[1]	60.83 (8.81)	48.88 (8.14)	<0.001	<0.001
Dissimulation[2]	76.17 (18.09)	52.25 (6.44)	<0.001	<0.001
Defensiveness[2]	46.71 (14.08)	52.33 (9.33)	0.02	0.86
**Clinical Scales**				
Cognitive Impairment[1]	62.21 (14.06)	54 (9.57)	0.02	<0.001
Impulsiveness and Distraction[2]	57.67 (11.13)	49.92 (8.05)	<0.001	0.09
Delinquency[2]	57.92 (11.86)	47.96 (7.35)	<0.001	0.02
Family Dysfunction[2]	61.63 (12.67)	50.17 (10.51)	<0.001	0.008
Reality Distortion[2]	64.46 (17.57)	50.42 (12.99)	<0.001	0.01
Somatic Concern[2]	72.75 (16.13)	55.25 (12.91)	<0.001	0.001
Psychological Discomfort[2]	74.79 (12.97)	52.08 (12.95)	<0.001	<0.001
Social Withdrawal[2]	69.5 (14.64)	59.42 (11.98)	<0.001	0.1
Social Skills Deficits[2]	66 (11.6)	51.38 (13.08)	<0.001	0.002

Note: [1]Student's *t*-test / [2]Mann-Whitney.
All measurements were performed using an analysis of covariance (ANCOVA) and were controlled for IQ

Table S3 - Relationship between socioeconomic level, SRQ scores of the clinical group, IQ, and the mothers' assessment of their children's anxiety regarding the impact on the personality adjustment scales (PIC-2).

Clinical Scales (PIC-2)	Socioeconomic level		SRQ-20		IQ		Maternal MASC-2	
**Response Validity Scales**								
Inconsistency	-0.42	p < .01	0.39	p < .01	-0.58	p < .001	0.54	p < .001
Dissimulation	-0.31	p < .05	0.43	p < .01	-0.45	p < .01	0.74	p < .001
Defensiveness	0.24		-0.29	p < .05	0.51	p < .001	-0.45	p < .01
**Clinical Scales**								
Cognitive Impairment	-0.24		0.22		-0.51	p < .001	0.34	p < .05
Impulsivity and Distractibility	-0.26		0.22		-0.36	p < .05	0.45	p < .01
Delinquency	-0.28		0.23		-0.39	p < .01	0.45	p < .01
Family Dysfunction	-0.23		0.20		-0.38	p < .01	0.44	p < .01
Reality Distortion	-0.25		0.26		-0.43	p < .01	0.65	p < .001
Somatic Concern	-0.26		0.46	p < .01	-0.36	p < .05	0.66	p < .001
Psychological Discomfort	-0.35	p < .05	0.42	p < .01	-0.39	p < .01	0.69	p < .001
Social Withdrawal	-0.21		0.16		-0.33	p < .05	0.51	p < .001
Social Skills Deficits	-0.29	p < .05	0.23		-0.46	p < .01	0.46	p < .001

Note Pearson's correlation *r*
